# Predictors of preferred location of care in middle-aged individuals of a municipality in Japan: a cross-sectional survey

**DOI:** 10.1186/s12913-017-2293-1

**Published:** 2017-05-15

**Authors:** Kentaro Sugimoto, Masayo Kashiwagi, Nanako Tamiya

**Affiliations:** 1 School of Medicine, Yokohama-City University, 3-9 Fukuura, Kanazawa-ku, Yokohama, Kanagawa 236-0004 Japan; 2 Department of Health Services Research, Faculty of Medicine, University of Tsukuba, 1-1-1 Tenno-dai, Tsukuba, Ibaraki 305-8575 Japan

**Keywords:** Advance healthcare planning, Cross-sectional study, Healthcare delivery, LTC, Middle-aged

## Abstract

**Background:**

Japan has the highest rate of aging. To contain Long-Term Care (LTC) Insurance costs, the Japanese government is attempting to increase the proportion of individuals receiving home care services. However, demand for institutional care is increasing. These circumstances will decrease the sustainability of the LTC Insurance System. The objective of the present study was to identify predictors of the location of preference for LTC (home or a facility) in middle-aged individuals in a municipality.

**Methods:**

This was a cross-sectional study of middle-aged individuals (*n* = 906) in Tsukuba, Japan. Data primarily included individual or social factors (sex, age, household size, experience with caring for family, information sources about social services or municipality policy), factors about care prevention (self-reported health, efforts to promote health, motivation in life), and the preferred location of care. These variables were analysed with multiple logistic regression, using preferred location of care as the dependent variable.

**Results:**

A total of 693 respondents were analysed. Of these, 440 (63.5%) preferred home and 253 (36.5%) preferred a facility. The results of logistic regression analysis showed that a preference for facility was significantly associated with female sex, younger age, experience with caring for family, fewer information sources about social services or municipality policies, selecting ‘go to culture lessons/study to satisfy interests’, and not selecting ‘spending time happily with family’ under motivation in life.

**Conclusions:**

To support the selection of receiving home care services, municipalities must consider improving policies that reduce the burdens of present middle-aged caregivers, and promote the provision of care service information from multiple sources.

## Background

Aging is progressing worldwide, resulting in a growing elderly population that creates an unavoidable challenge for most developing and developed countries [1–3]. Japan, the most rapidly aging country, has a 26.7% aging rate, and the number of individuals who need Long-Term Care (LTC) increased 1.5 times over the past 10 years. This number will greatly increase from around 2025, as individuals born during the baby boom from 1947–49, reach the age ≥ 75 years [4]. The growth of the care-needs population from 2025 and beyond will have an unprecedented impact on the Japanese LTC system.

LTC has typically been provided to the elderly of Japan in hospitals. However, to contain medical costs, the government changed the care location from hospital to the home or an aged care facility, with the introduction of the LTC Insurance system in 2000. The ultimate goal of the Japanese government is to increase the proportion of individuals who receive care service at home in proportion to the increasing number who need care. This has financial benefits. Home care services incur lower LTC Insurance costs than institutional care services. Hence, an increase in homecare service users would enhance insurance sustainability [5]. Conversely, an increase in institutional care use would threaten it. To promote home care service usage, the government implemented policies such as increasing the number of home care agencies and of homecare staff. In addition, the government implemented preventive care approaches for middle-aged and older residents, such as activities for health promotion or programs providing opportunities to motivate participation in community life, to promote home care [6, 7].

However, the demand for LTC services is not consistent with the aim of these policies. Although the number of institutional care services users is 900,000 and the proportion is about 20% of all LTC Insured persons, there were another 524,000 people who were waiting for admission to nursing homes in 2014 [8]. The number of people has increased from 421,000 people in 2011, while the number of nursing homes is also increasing. This data indicates demand for facility care is increasing, although the government is promoting homecare. Insurers of LTC, that is, municipalities, need to identify predictors of preferred location of care in current middle-aged individuals (e.g. a facility or home). The findings would clarify what makes people prefer institutional care, and what prevents it, and enhance the sustainability of LTC insurance from 2025 and beyond.

Several studies have focused on predictors of preferred care location [9–11]; however, few studies have contributed to promoting home care usage from 2025. A cross-sectional study analysed the predictors in community-dwelling individuals, including middle-aged adults (aged 40–79) [10]. The results showed that younger age, female sex, having concerns about home care (e.g. being unable to adequately respond to sudden changes, and a heavy family burden) were associated with a preference for non-home care. Another study explored the predictors of middle-aged individuals’ (aged 40–64) preferred aging location (home or another location) within the context of the following scenarios: subjects become bedridden or have a disability that renders them unable to walk outside alone, without being limited to a certain disease [11]. This study reported that female sex, living alone/with only one family member, and having fewer community associations were associated with a preference for a location other than the home, in both scenarios. These results suggest that policies to reduce the burden of family caregiving or empower community relationships would be helpful to encourage home care services. However, the first study included subjects who were already elderly, and the second study had a low response rate (24.6%), and included subjects who preferred a relative’s house in the non-home group, although insured persons who lived in a relative’s house would also receive home care services.

Therefore, we completed an investigation of preferred location of care among middle-aged individuals in a municipality with the objective of exploring predictors of preferred location of care. The present findings can also provide useful information to other municipalities in Japan, as well as to other aging countries that utilize a LTC Insurance System.

## Methods

### Study setting and participants

The present study analysed the survey data that were gathered to assist in formulating municipal insured LTC service plans in Tsukuba, Japan. In February 2011, the municipality mailed a self-administered questionnaire to a random sample (*N* = 2,000) of the population, aged 40–64 years, the generation that includes baby boomers.

Tsukuba is located approximately 100 km east of Tokyo, and has a population of approximately 214,590. The elderly population rate (aged ≥ 65 years) in October 2010 was 15.8%, which was lower than Japan’s national average (aged ≥ 65 years, 22.8%) [12]. The middle-aged population rate (aged 40–64 years) was 31.3% in Tsukuba and 33.7% nationwide [12]. Of all individuals who receive care, the percentage who received home care was 79.0% in Tsukuba and 79.6% nationwide [13]; thus, the latter two rates in Tsukuba are similar to Japan’s national average.

### Questionnaire

#### Preferred location of LTC within the context of subjects’ LTC needs

The Japanese LTC Insurance system provides two types of care services for the elderly: home care and institutional care [14, 15]. The home care services include the following services: home-visit nursing/bathing/rehabilitation, day-care service, short-stay care, etc. However, the formal home care services are insufficient for elderly people living alone. In fact, about 80% of the elderly who need LTC receive informal daily supportive care from their families, while there are the above formal care services [16]. There are elderly people who move to other houses (such as a relative’s house) to receive informal care. Additionally, institutional care is provided at aged care facilities such as nursing homes [15]. Considering this background, the present study asked participants to select a preferred location of LTC within the context of their LTC needs from the following four items: receiving care in your home, including another house (such as a family member’s house); admission to facilities such as a nursing home for LTC; other locations; or unsure.

#### Individual factors

Individual factors included the following items: sex, age, disease (treatment receipt or subsequent effects: disease; no disease), and occupation (full-time employment, part-time employment, self-employment, no occupation). These items were asked to assess associations between LTC preference and sociodemographic characteristics.

#### Social factors

Social factors included the following items: household size (living alone, married couples, 2- or 3-generation families living together, and others), number of family members, and current relationship with neighbours (house visits, talking if we meet, only greeting, almost no relationship) assessed associations with family/community associations. Furthermore, we included experience with caring for family (experience, no experience) and information sources about social services or municipality policy (municipality’s public relations magazine, friends, television, personal computer [multiple answers allowed]), as family-care experience or knowledge could affect future preference [10].

#### Factors about care prevention

The Japanese government and municipalities (insurers of LTC Insurance) take various preventive care approaches for enabling residents to continue living in the home [6, 7]. One of these is that municipalities organize health promotion programs, such as exercise class, nutrition education, etc. [6]. They also have programs that may help people finding their personal motivation in life. The projects include lessons to create hobbies (such as learning musical instruments, calligraphy, photography etc.), providing jobs or volunteer activities, providing meeting spaces to promote community association [6, 7]. The present study asked respondents the following questions to assess whether the preventive care approaches will affect preferred location of care: self-reported health (very good, good, not good, poor), efforts to promote health and prevent disease (effort made, no effort made), motivation in life (have motivation in life: hobby, job, sports, go to culture lessons/study to satisfy interests, volunteer work, spending time happily with family, friends and neighbours, others [multiple answers allowed]; no motivation in life), knowledge of municipality’s care prevention policy (well informed, a little knowledge, not well informed, no knowledge).

The selected questionnaire items were based on previous studies and discussions with a municipal expert panel. The panel consisted of policymakers and the following 20 members: insured persons, representatives from associations of healthcare or public aid providers, persons who engaged in the provision of LTC services, and persons with relevant knowledge and experience.

A formal agreement between one author (TN) and Tsukuba city was established for using the survey data. Approval to complete the study was obtained from the University of Tsukuba Ethical Committee.

### Statistical analysis

First, the subjects were categorized into two groups based on a preference for home or a facility. Responses of ‘prefer other location for LTC’ and ‘unsure’ were excluded. We then conducted bivariate analyses to examine whether each variable was associated with the preferred location of care. To examine dichotomous independent variables, *χ* ^2^ and Fisher’s exact tests were utilized. A Wilcoxon rank sum test was used for continuous independent variables. Next, we performed a multiple logistic regression analysis as a multivariate analysis and calculated the odds ratios and 95% confidence intervals. Variables were included in the model if an association had a *p*-value < 0.20, following confirmation for multicollinearity. Because previous studies have reported an association with sex, age, household size, self-reported health, and experience with caring for family [9–11], these variables were included in the model, regardless of the current association. The goodness of fit for the model was confirmed using the Hosmer-Lemeshow test. All analyses were performed using SAS version 9.4 software (SAS Institute, Cary, North Carolina, USA).

## Results

### Study sample

Of the mailed 2,000 questionnaires, 906 responses were received (response rate, 45.3%). Questionnaires of the following respondents were excluded: those aged ≥ 65 years (*n* = 4), those who did not provide a preferred location of care (*n* = 17), and those who responded ‘prefer other location for LTC’ (*n* = 32) or ‘unsure’ (*n* = 160). Ultimately, 693 respondents were included in the analysis.

### Predictors of preferred location of care in middle-aged individuals

The preferred location of care is presented in Table 1. In total, 440 (63.5%) and 253 (36.5%) subjects were categorized into the home and facility preference groups.

**Table 1:** Preferred location of care (*n* = 693)

Preferred location		*n*	(%)
Home	Receiving care in your home: including another house (such as a family member’s house)	440	(63.5)
Facility	Admitting to facilities such as a nursing home for LTC	253	(36.5)

The bivariate analysis results are shown in Table 2. The variables with a *p*-value < 0.20 (i.e. sex, age, household size, type of occupation [e.g. part-time and self-employment], relationship with neighbours, kind of motivation in life [hobby, go to culture lessons/study to satisfy interests, volunteer work, spending time happily with family], knowledge of municipality's care prevention policy, number of information sources, types of sources [television]), and the variables that were significantly associated with preferred location of care in previous studies (e.g. self-reported health, experience with caring for family), were used as control variables in the multiple regression analysis.

**Table 2:** Factors associated with preferred location of care (*n* = 693)

		Preferred location				*p*-value
		Home (*n* = 440)		Facility (*n* = 253)		
Sex	Men	187	42.9	76	30.3	<0.01^**^
	Women	249	57.1	175	69.7	
	Missing	4		2		
Age	Median (25–75%)	53	(47–60)	50	(45–58)	<0.01^**a^
	Missing	10		3		
Household size	Living alone	15	3.5	16	6.5	0.17
	Married couples	85	19.8	56	22.6	
	2- or 3-generation families living together	282	65.6	155	62.5	
	Others	48	11.2	21	8.5	
	Missing	10		5		
Number of family members	Median (25–75%)	3	(3–5)	3	(2–4)	0.33﻿^a﻿^
	Missing	28		23		
Disease (receiving treatment or experiencing subsequent effects)	Have disease	201	54.2	117	53.9	0.95
	No disease	170	45.8	100	46.1	
	Missing	69		36		
Self-reported health	Very good/good	397	90.9	224	88.9	0.41
	Not good/poor	40	9.2	28	11.1	
	Missing	3		1		
Occupation	Employment/Self-employment	344	78.7	190	75.4	0.31
	No occupation	93	21.3	62	24.6	
	Missing	3		1		
Type of occupation	Full-time employment	163	37.3	87	34.5	0.47
	No full-time employment	274	62.7	165	65.5	
	Missing	3		1		
	Part-time employment	103	23.6	71	28.2	0.18
	No part-time employment	334	76.4	181	71.8	
	Missing	3		1		
	Self-employment	57	13.0	20	7.9	0.04^*^
	No self-employment	380	87.0	232	92.1	
	Missing	3		1		
Experience with caring for family	Have experience	172	40.6	99	41.8	0.76
	No experience	252	59.4	138	58.2	
	Missing	16		16		
Relationship with neighbours	House visits/talking if met	218	53.0	107	45.7	0.07
	Only greeting/almost no relationship	193	47.0	127	54.3	
	Missing	29		19		
Effort to promote health and prevent disease	Make an effort	400	92.4	233	93.2	0.69
	Do not make an effort	33	7.6	17	6.8	
	Missing	7		3		
Motivation in life	Have motivation in life	416	97.4	236	96.3	0.42
	No motivation in life	11	2.6	9	3.7	
	Missing	13		8		
Kind of motivation in life	Hobby	163	38.2	80	32.7	0.15
	No hobby	264	61.8	165	67.4	
	Missing	13		8		
	Job	85	19.9	47	19.2	0.82
	No job	342	80.1	198	80.8	
	Missing	13		8		
	Sports	125	29.3	62	25.3	0.27
	No sports	302	70.7	183	74.7	
	Missing	13		8		
	Go to culture lessons/study to satisfy interests	107	25.1	95	38.8	<0.01^**^
	Not go to culture lessons/study to satisfy interests	320	74.9	150	61.2	
	Missing	13		8		
	Volunteer work	62	14.5	45	18.4	0.19
	Not volunteer work	365	85.5	200	81.6	
	Missing	13		8		
	Spending time happily with family	209	49.0	95	38.8	0.01^*^
	Not spending time happily with family	218	51.1	150	61.2	
	Missing	13		8		
	Friends and neighbours	151	35.3	77	31.4	0.30
	No friends and neighbours	276	64.6	168	68.6	
	Missing	13		8		
	Others	11	2.6	6	2.5	0.92
	No others	416	97.4	239	97.6	
	Missing	13		8		
Knowledge of municipality's care prevention policy	Well informed/a little knowledge	64	15.0	18	7.5	<0.01^**^
	Not well informed/No knowledge	362	85.0	222	92.5	
	Missing	14		13		
Number of information source	Median (25–75%)	2	(1–3)	2	(1–3)	<0.01^**a^
	Missing	7		5		
Types of information source	Municipality’s public relations magazine	318	73.4	175	70.6	0.42
	Other	115	26.6	73	29.4	
	Missing	7		5		
	Friends	68	15.7	38	15.3	0.89
	Not friends	365	84.3	210	84.7	
	Missing	7		5		
	Television	82	18.9	37	14.9	0.18
	Not television	351	81.1	211	85.1	
	Missing	7		5		
	Personal computer	44	10.2	28	11.3	0.64
	Not personal computer	389	89.8	220	88.7	
	Missing	7		5		

^*^ *p* < 0.05, ^**^ *p* < 0.01; ^a^Wilcoxon rank sum test, other variables: *χ* ^2^ test

The multiple logistic regression analysis results are shown in Table 3. In the multiple logistic regression analysis, preference for a facility as the location of care was significantly associated with the following six factors: female sex, younger age, experience with caring for family, selecting ‘go to culture lessons/study to satisfy interests’ and not selecting ‘spending time happily with family’ under kind of motivation in life, fewer information sources about social services or municipality policy. The goodness of fit for the model displayed a *p*-value of 0.78.

**Table 3:** Factors associated with the preferred location of care from multiple logistic regression analysis^a^ (*n* = 539)

		OR	95% CI	*p*-value
Sex^b^	Women	0.65	0.42–0.99	0.04^*^
Age (years)	Unit = 1 year	1.04	1.01–1.07	0.01^**^
Household size^c^	Married couples/2- or 3-generation families living together	1.59	0.71–3.56	0.26
Self-reported health﻿^d﻿^	Very good/good	0.99	0.51–1.92	0.98
Occupation	Self-employed	1.97	0.97–4.01	0.06
	Part-time employee	1.02	0.65–1.62	0.93
Experience of caring for family	Have experience	0.59	0.39–0.90	0.01^*^
Relationship with neighbours^e^	Go to each other’s house/Talk if we meet	1.22	0.82–1.80	0.33
Kind of motivation in life	Hobby	1.27	0.85–1.88	0.24
	Go to culture lessons/study to satisfy interests	0.56	0.37–0.85	0.01^**^
	Volunteer works	0.93	0.54–1.59	0.79
	Spending time happily with family	1.65	1.13–2.41	0.01^**^
Knowledge of municipality’s care prevention policy^f^	Know well/Know a little	1.62	0.83–3.13	0.16
Number of information sources	Unit = 1 source	1.20	1.02–1.42	0.03^*^
Types of information sources	Television	1.04	0.58–1.84	0.91

^a^Preference for home = 1, preference for facility = 0. ^b^Reference: men. ^c^Reference: living alone. ^d^Reference: not good/poor. ^e^Reference: only greeting/almost no relationship. ^f^Reference: not well informed/no knowledge

*p < 0.05, **p < 0.01; Hosmer-Lemeshow test *χ* ^2^ = 4.82, *p*-value = 0.78. *OR* odds ratio, *CI* confidence interval

## Discussion

The present study obtained the percentage of people who preferred the home or a facility as the location of care, and identified predictors of preferred location of care in middle-aged individuals. Although the percentage of preference for institutional care was similar to previous findings in another country (the present study: 36.5%, the previous study in the US: 35.0% [17]), the Japanese government needs to make further efforts to promote homecare, because younger age was associated with preference for institutional care. It is difficult to determine whether the association is due to a generation effect or an aging effect. However, considering the increased demand for institutional care, many of the current younger generation may select institutional care when they need LTC. Further examination of these findings will be necessary to aid in policy discussions that encourage the selection of receiving home care service, for those who want it, resulting in enhanced sustainability of LTC insurance.

Our study clarified that there was an association of ‘experience with caring for family’ with preference for institutional care. This finding indicates experience of caring makes middle-aged people prefer non-home care. The background to this may reflect certain characteristics of middle-aged caregivers. About 60% of middle-aged caregivers are daughters-in-law and daughters [16]. A previous study investigated about 3,500 Japanese people’s care burden using the eight-item short version of the Zarit Burden Interview (J-ZBI_8) [18], and the study reported that daughters-in-law and daughters had a heavier care burden than others (standardized score: 14, 13 respectively; the score exceeds the cut-off point of depression) [19]. In Japan, family care for the elderly has typically been the responsibility of women such as daughters-in-law or daughters. Cultural characteristics may make their burden too heavy. Another previous study found that family caregivers who have a job had a heavier care burden than those who are unemployed [20]. These people also account for about 60% of middle-aged caregivers [21]. These facts suggest that middle-aged caregivers have a heavy care burden. The circumstances may make middle-aged caregivers reluctant to become a burden on their family in the future.

To support the selection of receiving home care service, municipalities should reduce the caregiver burden of currently middle-aged individuals. This effort will make the experience with family care more positive. One study reported that the self-reported health of caregivers has not changed since LTC Insurance was introduced, even though reducing family care burden was one of its purposes [22]. Although the LTC Insurance system provides home care services that support elderly people who need care, family caregivers cannot receive any services [15]. Municipalities may have to provide not only home care services covered by the LTC insurance, but also additional care services for caregivers, such as health checks, mental health support, education for alleviation of care burden, supporting domestic tasks other than care [23], etc.

People having ‘multiple information sources about social services or municipality policy’ were more likely to prefer home care. Considering the association between concerns about home care (e.g. being unable to adequately respond to sudden changes) and non-home preference [10], increasing the number of information sources and subsequent knowledge of social services may alleviate concerns and support the selection of receiving home care service for those who want it. To realize this, the provision of care service information from multiple sources will also be necessary.

There was no association between self-reported health or effort to promote health, and preference for care location. These may not be factors in the preferences of middle-aged residents, because about 90% of the participants answered with ‘good health’ or ‘make an effort’. This suggests that health promotion policies cannot affect the preferences of middle-aged residents. However, these factors may be associated with homecare service use in the future, because a previous study clarified that self-reported good health is associated with homecare preference in the old-aged generation [9]. In addition, another study clarified that declining physical function will be factor in aged care facility admission [24]. Thus, health promotion policies will contribute to promoting homecare.

Additionally, more than 90% of participants had ‘motivation in life’, and it was not associated with preference of care location. A previous study found motivation in life had positive effects on physical function [25]. Considering the findings, policies to support people in finding motivation in life may also contribute to promoting homecare service usage in the future. The present study found that certain types of motivation in life were specifically associated with preference. As insurers cannot make residents to have the specific motivation, discussion focusing on personal characteristics of individuals who have these motivations in life is provided below.

First, ‘go to culture lessons/study to satisfy interests’ was associated with facility preference. A previous survey reported that middle-aged individuals willing to participate in social activities (such as going to culture lessons) tend to have no economic concerns, and wished to avoid becoming isolated elderly people [26]. These characteristics may enable middle-aged individuals’ preferences for institutional care. Second, ‘spending time happily with family’ was associated with home preference. A previous study focusing on preferences of elderly individuals [9] reported that ‘living with family’ was associated with a preference for home. The present study conducted a multivariate analysis that included ‘household size’, ‘number of family members’, and ‘spending time happily with family’. However, an association was observed only for the latter. This might be because most of the sample lived with spouse or others in this study. Alternatively, thinking that relationship with one’s family is good or having the prospect of living with them might have a stronger effect on preference than household size.

The present findings may prove useful for other municipalities in Japan, as well as for other countries, that will soon be facing similar situations with an aging population and the introduction of a LTC insurance system that requires the promotion of home care services.

Some limitations of the present study should be acknowledged. First, a major limitation of the present study was that it was conducted in only one Japanese municipality where the elderly population percentage was lower than the national average. This may have diminished the perception of respondents concerning care burden or concerns about receiving LTC. In a municipality in which aging is advancing more rapidly than Tsukuba city, the association of experience with caring for family or information sources of social services may be more pronounced. This means the actual percentage of preference for a facility is higher than the present result. If that is it, the municipality will need to make further efforts to encourage the selection of receiving home care services. Second, the response rate of the present study was low (45.3%). Considering that it may have been easier for individuals who already have a preferred care location to respond to this survey, the actual percentage of people who are ‘unsure’ of their preferred care location may be higher than the present results indicate. This means that the percentage of preferences might shift as these individuals make their decisions. The present findings only represent the future perceived wants of middle-aged individuals, and the percentage will be also affected by changes of preference as these individuals age. Considering these limitations, further studies that can observe shifts in preferences for location of care of the current middle-aged generation will be needed. Next, although the present study asked about preferred care location within the context of subjects’ LTC needs, level of disability was not assessed. Further research that provides multiple assessments of subjects with mild to heavy disabilities will be needed. Such research would clarify the needs of middle-aged people more clearly. Finally, since the design was cross-sectional, causality cannot be inferred from our results.

## Conclusions

In conclusion, to support the selection of receiving home care services, and to enhance the sustainability of the LTC insurance program from 2025 and beyond, it is necessary that municipalities improve policies to make experiences with family care more positive, reduce the burden of present middle-aged family caregivers, and promote the provision of care service information from multiple sources.

## Abbreviations

LTC: Long-term care
